# Prevalence of overweight misperception and weight control behaviors among normal weight adolescents in the United States

**DOI:** 10.1100/tsw.2006.70

**Published:** 2006-03-26

**Authors:** Kathleen S. Talamayan, Andrew E. Springer, Steven H. Kelder, Emmanuel C. Gorospe, Karen A. Joye

**Affiliations:** ^1^ Centers for Health Promotion and Prevention Research, School of Public Health, University of Texas Health Science Center, Houston, TX, USA; ^2^ School of Public Health, University of Nevada Las Vegas, USA

**Keywords:** adolescent behavior, weight perception, weight reduction, weight control behavior, United States

## Abstract

Weight perceptions and weight control behaviors have been documented with underweight and overweight adolescents, yet limited information is available on normal weight adolescents. This study investigates the prevalence of overweight misperceptions and weight control behaviors among normal weight adolescents in the U.S. by sociodemographic and geographic characteristics. We examined data from the 2003 Youth Risk Behavior Survey (YRBS). A total of 9,714 normal weight U.S. high school students were included in this study. Outcome measures included self-reported height and weight measurements, overweight misperceptions, and weight control behaviors. Weighted prevalence estimates and odds ratios were computed. There were 16.2% of normal weight students who perceived themselves as overweight. Females (25.3%) were more likely to perceive themselves as overweight than males (6.7%) (p < 0.05). Misperceptions of overweight were highest among white (18.3%) and Hispanic students (15.2%) and lowest among black students (5.8%). Females (16.8%) outnumbered males (6.8%) in practicing at least one unhealthy weight control behavior (use of diet pills, laxatives, and fasting) in the past 30 days. The percentage of students who practiced at least one weight control behavior was similar by ethnicity. There were no significant differences in overweight misperception and weight control behaviors by grade level, geographic region, or metropolitan status. A significant portion of normal weight adolescents misperceive themselves as overweight and are engaging in unhealthy weight control behaviors. These data suggest that obesity prevention programs should address weight misperceptions and the harmful effects of unhealthy weight control methods even among normal weight adolescents.

## INTRODUCTION

The preference for thinness in the mass media has a strong influence in shaping adolescents' concept of ideal body weight[1,2]. As adolescents have been found to be extremely self-conscious of their bodies[3], social pressures from the mass media may cause even normal weight adolescents to misperceive themselves as overweight. In the U.S. National Health and Nutrition Examination Survey (NHANES III), 42% of adolescents who considered themselves as overweight were actually of normal weight[4]. Based on studies[5,6] reporting the prevalence of unhealthy weight control practices among overweight adolescents, there is potential for normal weight adolescents who misperceive themselves as overweight also to engage in unhealthy weight control behaviors.

Unhealthy weight control behaviors such as fasting, purging, and use of diet pills may adversely affect adolescents' physical and psychosocial development. Unhealthy outcomes include the development of eating disorders[7,8], stress[9], other mental health problems, and impairment of cognitive abilities[10,11]. Despite the potential risks associated with unhealthy weight control behaviors, few studies have specifically examined this issue among normal weight adolescents.

This study reports the prevalence of overweight misperceptions and weight control behaviors among normal weight adolescents from the 2003 Youth Risk Behavior Survey (YRBS), a nationally representative survey of U.S. high school students. In addition to examining the prevalence of overweight misperceptions and weight control practices by several sociodemographic and geographic characteristics, this paper explores the association between overweight misperception and unhealthy weight control behaviors.

## METHODS

We conducted a secondary analysis of the cross-sectional data from the 2003 YRBS, a comprehensive school-based adolescent health survey that has been administered biennially in the U.S. since 1991 by the Centers for Disease Control and Prevention (CDC) with the aim of monitoring priority health-risk behaviors among youth. The YRBS is based on a three-stage cluster sample design to produce a nationally representative sample of high school students in grades 9 through 12. Participants completed a self-administered questionnaire that included items on various youth risk behaviors. Participation in the survey was anonymous and voluntary, and local parental permission procedures were followed. The specific sampling design and methods for the YRBS are described in greater detail elsewhere[12].

### Participants

There were 15,214 respondents from 158 public and private high schools participating in the study. The student and school response rate were 83 and 81%, respectively. For the purposes of the present analysis, we selected the 9,714 normal weight students. Normal weight status was based on the CDC Body Mass Index (BMI) percentile categories for age and sex[13]. A weighting factor was applied to each student record to adjust for nonresponse and for the varying probabilities of selection in order to ensure that the data equaled the total sample size and matched national population proportions.

### Measures

BMI was based on self-reported height and weight measurements and was obtained by asking, "How tall are you without your shoes on?" in feet, and "How much do you weigh without your shoes on?" in pounds. These values were converted to the metric system to derive the BMI (kilograms/square meter). Participants were then categorized as underweight, normal, at risk for overweight, and overweight. Values were based on the BMI-for-age and sex for pediatric population as per the CDC classifications with normal weight, defined as a BMI between the $5^{th}$ and $<85^{th}$ percentile.

Weight perception was assessed with the question, “How do you describe your weight?” Adolescents with normal BMI who responded “overweight” and “very overweight” were classified as those with misperception of overweight status. Information on different weight control behaviors was also collected. Trying to lose weight was determined by asking, “Which of the following are you trying to do about your weight?” with responses that included “lose weight” and “gain weight”. Exercise was assessed with the question, “During the past 30 days, did you exercise to lose weight or to keep from gaining weight?” Dieting was measured by asking, “During the past 30 days, did you eat less food, fewer calories, or foods low in fat to lose weight or to keep from gaining weight?” Unhealthy weight control behaviors that include fasting, taking diet medicines, and purging were determined with the following questions, respectively, “During the past 30 days, did you go without eating for 24 hours or more (also called fasting) to lose weight or to keep from gaining weight?”, “During the past 30 days, did you take any diet pills, powders, or liquids without a doctor’s advice to lose weight or to keep from gaining weight?”, “During the past 30 days, did you vomit or take laxatives to lose weight or to keep from gaining weight?” Fasting was defined as no food intake for more than 24 hours and purging was defined as either vomiting or taking laxatives to lose weight. Having at least one unhealthy weight loss behavior was described as reporting yes to any of the three weight control behaviors. The overweight misperception and weight control behavior variables were all dichotomized with a “yes” or “no” response. All outcomes were based on the YRBS questionnaire.

We examined the prevalence and distribution of overweight misperception and weight control behaviors by gender, grade level, race/ethnicity, geographic region, and metropolitan status. For this study, race/ethnicity comprised the following groups: white, black, Hispanics, and a combined category of “other” for those races/ethnicities with small sample size. This variable was based on response to the question, “How do you describe yourself?” Geographic region (Northeast, Midwest, South, or West) and metropolitan status (“urban”, “suburban”, and “rural”) were based on the location of the school attended by the student.

### Statistical Analysis

Analysis was performed using the STATA® version 9.0 statistical program that takes into account the complex sampling design of the YRBS. In describing the prevalence and distribution among the sample, we computed the weighted point prevalence estimates and standard errors of the overweight misperception and weight control behaviors for the sample as a whole and by the various sociodemographic variables described above. Logistic regression was performed to determine the bivariate associations between the principal study variables of interest: overweight misperception and weight control behaviors, with the sociodemographic and geographic variables. In assessing the association between overweight misperception and the weight control behaviors, we used logistic regression to compute the odds ratios while adjusting for gender and race/ethnicity. Differences between prevalence estimates were considered statistically significant at $p$-value < 0.05.

## RESULTS

In the 2003 YRBS sample, 71.0% of participants were classified as having normal body weight (BMI-for-age: $5^{th}$ to $<85^{th}$ percentile). Based on this normal weight classification, 9,714 students were included in this study. The sample was equally distributed across gender and grade level and was largely represented by white students (64.3%) and students residing in the suburban area (see Table 1).

**TABLE 1**
**Descriptive Characteristics of Normal Weight[a] U.S. High School Students by Sociodemographic and Geographic Characteristics, YRBS 2003**

| | | n | % |
|---|---|---|---|
| Total | Normal weight | 9,714 | 100 |
| Gender | Males | 4,716 | 48.5 |
| | Females | 4,998 | 51.5 |
| Grade level | 9th | 2,713 | 28.0 |
| | 10th | 2,578 | 26.6 |
| | 11th | 2,268 | 23.4 |
| | 12th | 2,135 | 22.0 |
| Race/ethnicity | Blacks | 1,211 | 12.5 |
| | Hispanics | 1,261 | 13.0 |
| | Whites | 6,242 | 64.3 |
| | Others[b] | 999 | 10.3 |
| Geographic region | Northeast | 2,206 | 22.7 |
| | Midwest | 2,535 | 26.1 |
| | South | 3,310 | 34.1 |
| | West | 1,663 | 17.1 |
| Metropolitan status | Urban | 2,492 | 25.7 |
| | Suburban | 5,058 | 52.1 |
| | Rural | 2,163 | 22.3 |

[a] Normal weight based on a BMI-for-age of 5th to <85th percentile.

[b] "Others" include American Indian or Alaskan Native, Asian, Native Hawaiian or Other Pacific Islander, Multiple Hispanic, Multiple non-Hispanic.

Of the normal weight respondents, 16.2% misperceived themselves as overweight (see Table 2). Moreover, 35.8% of the students indicated trying to lose weight, including those that did not perceive themselves to be overweight. Of normal weight students, 11.8% indicated at least one unhealthy weight control behavior. Among the unhealthy weight control behaviors, fasting was the most commonly practiced (11.6%), followed by taking diet medicine (7.4%) and purging (5.2%).

Normal weight adolescents who misperceived themselves as overweight varied by gender and race (see Table 2). Females (25.3%) were more likely to misperceive themselves as overweight than males (6.7%). There were more white (18.3%) and Hispanic (15.2%) students who misperceived themselves as overweight compared to black students (5.8%).

The misperception of being overweight tends to increase from the 9th to the 12th grade (see Table 2). The number of those trying to lose weight was greater among the higher grade levels. Parallel to this trend was the higher number of 11th and 12th grade students practicing at least one unhealthy weight control behavior compared to the 9th and 10th grade students. This pattern is best seen in the increasing proportion of students who reported taking dieting pills from 9th to 12th grade.

Females persistently outnumbered males in practicing weight control behaviors (see Table 2). Females were approximately three times as likely to exercise (OR = 2.6, 95% CI: 2.3–2.9) and five times as likely to diet (OR = 4.8, 95% CI: 4.0–5.7) than males (data not shown in tables). Unhealthy weight control behaviors (fasting, taking diet medicines, and purging) were also more common in females than in

**TABLE 2**
**Prevalence of Overweight Misperception and Weight Control Behaviors by Sociodemographic and Geographic Characteristics, YRBS 2003**

| Characteristic | Perceived Themselves as Overweight % (CI) | Were Trying to Lose Weight %(CI) | Exercised to Lose Weight or to Keep from Gaining Weight % (CI) | Ate Less Food, Fewer Calories, or Foods Low in Fat to Lose Weight or to Keep from Gaining Weight % (CI) | Went Without Eating for ≥24 h to Lose Weight or to Keep from Gaining Weight % (CI) | Took Diet Medicines to Lose Weight or to Keep from Gaining Weight % (CI) | Vomited or Took Laxatives to Lose Weight or to Keep from Gaining Weight % (CI) | At Least 1 Unhealthy Weight Control Behavior % (CI) |
|---|---|---|---|---|---|---|---|---|
| Total | 16.2 (±1.5) | 35.8 (±2.3) | 54.6 (±3.0) | 36.8 (±2.0) | 11.6 (±1.3) | 7.4 (±1.5) | 5.2 (±1.3) | 11.8 (±2.5) |
| Gender | | | | | | | | |
| Males | 6.7 (±1.0) | 15.5 (±2.1) | 42.7 (±2.8) | 19.2 (±2.3) | 6.3 (±1.2) | 4.6 (±1.7) | 2.6 (±1.1) | 6.8 (±1.6) |
| Females | 25.3 (±3.0) | 54.8 (±4.0) | 65.8 (±3.8) | 53.4 (±2.9) | 16.6 (±2.0) | 10.0 (±1.7) | 7.6 (±1.9) | 16.8 (±1.9) |
| Grade Level | | | | | | | | |
| 9th | 15.3 (±3.0) | 33.5 (±3.1) | 55.6 (±4.2) | 34.5 (±2.7) | 12.4 (±2.4) | 6.6 (±2.3) | 4.8 (±1.9) | 11.8 (±2.5) |
| 10th | 15.7 (±2.1) | 36.2 (±3.6) | 56.1 (±4.8) | 37.7 (±4.5) | 12.1 (±2.0) | 6.2 (±1.8) | 6.2 (±2.0) | 10.9 (±1.9) |
| 11th | 15.8 (±2.0) | 36.0 (±2.9) | 53.4 (±3.9) | 36.4 (±2.8) | 12.3 (±2.3) | 7.9 (±2.3) | 5.1 (±1.4) | 12.0 (±2.0) |
| 12th | 18.7 (±2.1) | 38.0 (±3.1) | 53.0 (±4.5) | 39.2 (±3.3) | 9.1 (±2.0) | 9.2 (±1.6) | 4.7 (±1.2) | 12.8 (±2.4) |
| Race | | | | | | | | |
| Blacks | 5.8 (±1.1) | 19.7 (±2.2) | 39.6 (±3.6) | 20.9 (±2.5) | 9.7 (±1.6) | 2.8 (±1.3) | 3.6 (±1.3) | 10.2 (±1.5) |
| Hispanics | 15.2 (±3.8) | 39.6 (±4.2) | 54.7 (±4.7) | 36.8 (±4.0) | 12.5 (±2.2) | 7.6 (±3.8) | 5.8 (±2.8) | 13.6 (±3.0) |
| Whites | 18.3 (±2.0) | 37.9 (±3.1) | 57.1 (±4.0) | 39.8 (±2.6) | 11.3 (±1.7) | 8.4 (±2.0) | 5.2 (±1.6) | 11.4 (±1.5) |
| Others[a] | 17.3 (±4.0) | 37.1 (±6.0) | 57.1 (±7.3) | 37.7 (±4.5) | 14.5 (±3.3) | 6.1 (±2.5) | 6.5 (±2.8) | 13.8 (±3.4) |
| Geographic Region | | | | | | | | |
| Northeast | 16.9 (±1.2) | 35.5 (±5.0) | 51.7 (±8.8) | 38.0 (±2.2) | 12.0 (±2.9) | 8.9 (±5.0) | 7.3 (±4.0) | 12.0 (±4.2) |
| Midwest | 17.8 (±4.4) | 40.1 (±3.8) | 59.4 (±2.8) | 42.0 (±4.2) | 12.5 (±1.3) | 6.9 (±1.6) | 4.6 (±1.2) | 12.8 (±1.9) |
| South | 14.1 (±1.3) | 32.2 (±3.2) | 51.3 (±2.9) | 31.1 (±2.2) | 11.0 (±1.7) | 6.2 (±1.4) | 4.3 (±1.1) | 10.9 (±1.6) |
| West | 17.4 (±3.3) | 36.6 (±5.8) | 57.8 (±5.8) | 39.1 (±4.2) | 11.0 (±3.7) | 8.6 (±5.0) | 5.3 (±4.3) | 11.8 (±2.6) |
| Metropolitan Status | | | | | | | | |
| Urban | 14.9 (±3.5) | 31.5 (±5.0) | 50.7 (±5.6) | 32.0 (±3.4) | 10.7 (±2.2) | 6.7 (±3.1) | 5.8 (±2.8) | 9.7 (±1.9) |
| Suburban | 16.7 (±1.3) | 37.6 (±1.9) | 57.1 (±2.3) | 37.6 (±2.3) | 12.1 (±1.8) | 6.8 (±1.3) | 4.5 (±1.5) | 12.4 (±1.6) |
| Rural | 16.8 (±2.5) | 36.4 (±7.9) | 53.3(±11.1) | 40.8 (±3.7) | 11.6 (±2.4) | 9.6 (±4.3) | 6.1 (±3.7) | 12.8 (±3.3) |

Abbreviations: CI - 95% confidence interval.

[a] "Others" include American Indian or Alaskan Native, Asian, Native Hawaiian or Other Pacific Islander, Multiple Hispanic, Multiple non-Hispanic.

males (see Table 2). For example, females were approximately three times as likely to report at least one weight control behavior compared to males (OR = 2.8, 95% CI: 2.1–3.7; data not shown in tables).

White and Hispanic students were twice as likely to engage in physical activity (OR = 1.8, 95% CI: 1.4–2.4 and OR = 2.0, 95% CI 1.6–2.6, respectively) and diet (OR = 2.2, 95% CI: 1.7–2.8 and OR = 2.5, 95% CI: 2.1–3.0, respectively) as compared to black students (data not shown in tables). These same ethnic groups were also approximately thrice as likely to attempt losing weight (OR = 2.7, 95% CI 2.2–3.3 and OR = 2.5, 95% CI: 2.1–3.0, respectively) and to take diet pills (OR = 2.9, 95% CI: 1.9–4.4 and OR = 3.2, 95% CI: 1.9–5.5, respectively) than black students (data not shown in tables). There was no significant difference in the prevalence of having at least one unhealthy weight control behavior among the ethnic groups.

In addition, there were no significant differences observed in overweight misperception and weight control behaviors by grade level, geographic region, or metropolitan status.

The associations between overweight misperception and the different weight control behaviors were determined while controlling for gender and race/ethnicity (see Table 3). Among the normal weight students, those who perceived themselves as overweight were three times as likely to engage in each of the unhealthy weight control behaviors examined as those who did not perceive themselves as overweight.

**TABLE 3**
**Odds Ratios of Overweight Perception and Weight Control Behaviors of U.S. Normal Weight Students, YRBS 2003**

| | Exercised to Lose Weight or to Keep from Gaining Weight | Ate Less Food, Fewer Calories, or Foods Low in Fat to Lose Weight or to Keep from Gaining Weight | Went Without Eating for ≥24 h to Lose Weight or to Keep from Gaining Weight | Took Diet Medicines to Lose Weight or to Keep from Gaining Weight | Vomited or Took Laxatives to Lose Weight or to Keep from Gaining Weight | At Least 1 Unhealthy Weight Control Behavior |
|---|---|---|---|---|---|---|
| Perceived themselves as overweight[a] | OR (CI 95%) | OR (CI 95%) | OR (CI 95%) | OR (CI 95%) | OR (CI 95%) | OR (CI 95%) |
| Crude OR | 4.1 (3.5–4.9) | 6.8 (5.6–8.1) | 3.8 (3.0–4.7) | 4.1 (2.9–5.7) | 3.9 (2.6–6.0) | 3.3 (2.5–4.3) |
| Adjusted OR[b] | 3.0 (2.5–3.6) | 4.6 (3.8–5.7) | 3.0 (2.4–3.7) | 3.1 (2.3–4.3) | 3.0 (2.0–4.5) | 2.6 (2.0–3.4) |

Abbreviations: OR, odds ratio; CI, confidence interval.

[a] Referent group = students who did not perceive themselves as overweight.

[b] Adjusted for gender and ethnicity.

## DISCUSSION

This study investigated the prevalence of overweight misperception and weight control practices of normal weight adolescents in the U.S. We found that a significant percentage of normal weight high school students (16.2%) misperceived themselves as overweight and that more than a third (35.8%) reported trying to lose weight in spite of their normal BMI. Of particular concern, 11.8% of students reported practicing at least one unhealthy weight control behavior such as purging or fasting in the past 30 days. These findings suggest that normal weight adolescents may be at risk for adverse health effects related to unhealthy weight control behaviors.

Similar to past studies, this study observed several important gender[4,14,15,16,17] and ethnic[4,17] differences in overweight misperceptions. Our finding of a higher prevalence of overweight misperceptions among female adolescents may be a result of a greater concern with body image as compared to their male counterparts[15,18,19]. In spite of the large gender difference in the prevalence of overweight misperception and unhealthy weight control behaviors, there is nevertheless a significant number of male students (>6%) who reported overweight misperceptions and engagement in more than one unhealthy weight control behavior.

With regard to ethnic differences in overweight misperceptions, our findings are consistent with the results of the NHANES III and the 1990 North Carolina YRBS report, which found white females to be more inclined to misperceive themselves as overweight[4,20]. This study also found African-American adolescents with normal weight to be the least likely to perceive themselves as overweight. Even as children, African-Americans are most satisfied with their body weight compared to whites and other racial minorities[21]. Studies have suggested lesser social and cultural pressure for blacks to be thin as compared to their white and Hispanic counterparts[22,23].

Having distorted weight perception may set unrealistic goals and unhealthy weight control behaviors among adolescents[17]. There is some evidence to suggest that body weight misperception may be more influential than actual weight status in enticing adolescents to practice weight control[4,24]. This study supports the hypothesis that students who misperceive themselves as overweight are more likely to engage in various unhealthy weight control behaviors.

Of particular concern was our finding of a substantial prevalence of fasting (11.6%) and diet pill use (7.4%) among our normal weight sample. Unaware of their ill effects, adolescents might consider these behaviors as the fastest and easiest course to lose weight. Fasting has been found in previous research to be highest among younger adolescents[25]. Our results also suggest that fasting is higher among those in the first 3 years of high school, with a slight decrease by 12$^{th}$ grade. The intake of diet pills, on the other hand, was more prevalent among higher grade levels. As exercise and healthy dieting remained constant throughout the grade levels in this study, the increase in diet pill consumption may reflect an increasing pressure to lose weight through the fastest means possible, especially for older adolescents who may have greater access to diet pills compared to their younger schoolmates. Concern for the use of diet pills is underscored when comparing the prevalence of diet pill use among adolescents in previous studies. In the 1990 YRBS, the use of dieting pills was only 4% among females and 2% among males[22]. In our data, 10% of females and 4.6% of males have used dieting pills. While results from the 1999 YRBS showed higher prevalence of abnormal weight control behavior in the Midwestern and southern states[26], we did not find significant differences in the prevalence of unhealthy weight control practices by geographic region and metropolitan status. The lack of association between the prevalence of weight control behaviors and place of residence found in this study may suggest the popularity of unhealthy weight control across the U.S. that results from widespread exposure of media among U.S. adolescents.

An important limitation of this study was the determination of the BMI status and classification of normal weight adolescents based on self-reported height and weight. As adolescents have been found to overreport their height and underreport their weight[27], it is possible that some students who were actually overweight were misclassified as normal weight. The potential misclassification of overweight students as normal weight may have led to an overestimate of the prevalence of overweight misperceptions and weight control behaviors among this sample.

## CONCLUSIONS

In conclusion, a significant portion of normal weight adolescents misperceived themselves as overweight and are practicing at least one unhealthy weight loss behavior. This study’s findings also suggest that overweight misperception is associated with unhealthy weight control behaviors. With the increasing prevalence of childhood obesity[28,29], our results recommend the need for a holistic perspective in addressing healthy weight issues among adolescents. Many program implementers working with the childhood obesity issue may be tempted to focus their efforts on strategies for reducing weight among overweight adolescents, with little attention given to the majority of the adolescents who, in spite of their normal weight, may be struggling to maintain or to lose further weight. Childhood obesity prevention programs need to include educational components that address the risks of unhealthy weight control behaviors among adolescents, regardless of BMI status, in addition to the benefits of healthy weight maintenance behaviors.

## ACKNOWLEDGMENTS

The authors would like to acknowledge Bob Addy, Research Associate at the Centers for Health Promotion and Prevention Research, University of Texas-Houston School of Public Health, and Julia

Sanders, Graduate Research Assistant at the Human Nutrition Center, University of Texas-Houston School of Public Health, for their insightful guidance with the analysis of the YRBS data. Preparation of this manuscript was made possible in part by the National Cancer Institute/NIH Grant# 2R25CA57712-11 which supports Dr. Springer's Postdoctoral Fellowship as well as the Cancer Prevention and Control Training Program in the Centers for Health Promotion and Prevention Research, University of Texas-Houston School of Public Health.

## References

1. Rodin, J. (1993) Cultural and psychosocial determinants of weight concerns. *Ann. Intern. Med.* **119(7 Pt 2),** 643–645.
2. Field, A.E., Camargo, C.A., Jr., Taylor, C.B., et al. (1999) Relation of peer and media influences to the development of purging behaviors among preadolescent and adolescent girls. *Arch. Pediatr. Adolesc. Med.* **153(11),** 1184–1189.
3. Phelps, L., Johnston, L., Jimenes, D., et al. (1993) Figure preference, body dissatisfaction, and body distortion in adolescence. *J. Adolesc. Res*. **8**, 297–311.
4. Strauss, R.S. (1999) Self-reported weight status and dieting in a cross-sectional sample of young adolescents: National Health and Nutrition Examination Survey III. *Arch. Pediatr. Adolesc. Med*. **153(7),** 741–747.
5. Boutelle, K., Neumark-Sztainer, D., Story, M., et al. (2002) Weight control behaviors among obese, overweight, and nonoverweight adolescents. *J. Pediatr. Psychol*. **27(6),** 531–540.
6. Neumark-Sztainer, D., Story, M., Falkner, N.H., et al. (1999) Sociodemographic and personal characteristics of adolescents engaged in weight loss and weight/muscle gain behaviors: who is doing what? *Prev. Med*. **28(1),** 40–50.
7. Killen, J.D., Taylor, C.B., Hayward, C., et al. (1996) Weight concerns influence the development of eating disorders: a 4-year prospective study. *J. Consult. Clin. Psychol*. **64(5),** 936–940.
8. Killen, J.D., Taylor, C.B., Hayward, C., et al. (1994) Pursuit of thinness and onset of eating disorder symptoms in a community sample of adolescent girls: a three-year prospective analysis. *Int. J. Eat. Disord*. **16(3),** 227–238.
9. Rosen, T. and Howel, D. (1990) Life stress, psychological symptoms and weight reducing behavior in adolescent girls: a prospective analysis. *Int. J. Eat. Disord*. **9**, 7–26.
10. Rogers, P.J. and Green, M.W. (1993) Dieting, dietary restraint and cognitive performance. *Br. J. Clin. Psychol*. **32 (Pt 1),** 113–116.
11. Stice, E., Cameron, R.P., Killen, J.D., et al. (1999) Naturalistic weight-reduction efforts prospectively predict growth in relative weight and onset of obesity among female adolescents. *J. Consult. Clin. Psychol*. **67(6),** 967–974.
12. Centers for Disease Control and Prevention (2003) National School-Based Youth Risk Behavior Survey: Public-Use Data Document. Retrieved on October 24, 2005 from http://www.cdc.gov/HealthyYouth/yrbs/data/2003/yrbs2003codebook.pdf
13. Centers for Disease Control and Prevention (2005) Body Mass Index: BMI for Children and Teens. Retrieved on October 24, 2005 from http://www.cdc.gov/nccdphp/dnpa/bmi/bmi-for-age.htm
14. Childress, A.C., Brewerton, T.D., Hodges, E.L., et al. (1993) The Kids' Eating Disorders Survey (KEDS): a study of middle school students. *J. Am. Acad. Child Adolesc. Psychiatry* **32(4),** 843–850.
15. Gustafson-Larson, A.M. and Terry, R.D. (1992) Weight-related behaviors and concerns of fourth-grade children. *J. Am. Diet. Assoc*. **92(7),** 818–822.
16. Field, A.E., Camargo, C.A., Jr., Taylor, C.B., et al. (1999) Overweight, weight concerns, and bulimic behaviors among girls and boys. *J. Am. Acad. Child Adolesc. Psychiatry* **38(6),** 754–760.
17. Felts, W.M., Parrillo, A.V., Chenier, T., et al. (1996) Adolescents' perceptions of relative weight and self-reported weight-loss activities: analysis of 1990 YRBS (Youth Risk behavior Survey) national data. *J. Adolesc. Health* **18(1),** 20–26.
18. Thelen, M., Powell, A., Lawrence, C., et al. (1992) Eating and body image concern among children. *J. Clin. Psychol*. **21**, 41–46.
19. Feldman, W., Feldman, E., and Goodman, J.T. (1988) Culture versus biology: children's attitudes toward thinness and fatness. *Pediatrics* **81(2),** 190–194.
20. Felts, M., Tavasso, D., Chenier, T., et al. (1992) Adolescents' perceptions of relative weight and self-reported weight loss activities. *J. Sch. Health* **62(8),** 372–376.
21. Welch, C., Gross, S.M., Bronner, Y., et al. (2004) Discrepancies in body image perception among fourth-grade public school children from urban, suburban, and rural Maryland. *J. Am. Diet. Assoc*. **104(7),** 1080–1085.
22. Serdula, M.K., Collins, M.E., Williamson, D.F., et al. (1993) Weight control practices of US adolescents and adults. *Ann. Intern. Med*. **119(7 Pt 2),** 667–671.
23. Kumanyika, S.K. (1993) Special issues regarding obesity in minority populations. *Ann. Intern. Med.* **119(7 Pt 2),** 650–654.
24. Brener, N.D., Eaton, D.K., Lowry, R., et al. (2004) The association between weight perception and BMI among high school students. *Obes. Res*. **12(11),** 1866–1874.
25. Maloney, M.J., McGuire, J., Daniels, S.R., et al. (1989) Dieting behavior and eating attitudes in children. *Pediatrics*

**84(3),** 482–489.

26. Forman-Hoffman, V. (2004) High prevalence of abnormal eating and weight control practices among U.S. high-school students. *Eat. Behav*. **5(4),** 325–336.
27. Brener, N.D., McManus, T., Galuska, D.A., et al. (2003) Reliability and validity of self-reported height and weight among high school students. *J. Adolesc. Health* **32(4),** 281–287.
28. Hedley, A.A., Ogden, C.L., Johnson, C.L., et al. (2004) Prevalence of overweight and obesity among US children, adolescents, and adults, 1999–2002. *JAMA* **291(23),** 2847–2850.
29. Strauss, R.S. and Pollack, H.A. (2001) Epidemic increase in childhood overweight, 1986–1998. *JAMA* **286(22),** 2845–2848.

## BIOSKETCHES

**Kathleen S. Talamayan, MD, MPH**, Centers for Health Promotion and Prevention Research, School of Public Health, University of Texas Health Science Center at Houston, Houston, TX.

**Andrew E. Springer, DrPH**, Centers for Health Promotion and Prevention Research, School of Public Health, University of Texas Health Science Center at Houston, Houston, TX. E-mail: andrew.e.springer@uth.tmc.edu.

**Steven H. Kelder, MPH, PhD**, Centers for Health Promotion and Prevention Research, School of Public Health, University of Texas Health Science Center at Houston, Houston, TX.

**Emmanuel C. Gorospe, MD**, The Children's Nephrology Clinic, 3201 S. Maryland Pkwy #606, Las Vegas, NV 89109; Tel: (702)743-7332/Fax: (702)639-1717. E-mail: GorospeE@unlv.nevada.edu

**Karen A. Joye, BA**, Centers for Health Promotion and Prevention Research, School of Public Health, University of Texas Health Science Center at Houston, Houston, TX.
